# Antitumor effect of TW-37, a BH3 mimetic in human oral cancer

**DOI:** 10.1186/s42826-019-0028-7

**Published:** 2019-12-04

**Authors:** Chi-Hyun Ahn, Won Woo Lee, Yun Chan Jung, Ji-Ae Shin, Kyoung-Ok Hong, Sujung Choi, Neeti Swarup, Jihoon Kim, Min-Hye Ahn, Minjung Jung, Sung-Dae Cho, Bohwan Jin

**Affiliations:** 1 Department of Oral Pathology, School of Dentistry and Dental Research Institute, Seoul National University, Seoul, 03080 Republic of Korea; 2 Laboratory Animal Center, CHA University, CHA Biocomplex, Sampyeong-dong, Seongnam, 13488 Republic of Korea; 3 Chaon, 301-3, 240, Pangyoyeok-ro, Bundang-gu, Seongnam, 13493 Republic of Korea

**Keywords:** TW-37, Apoptosis, Bcl-2, Oral cancer

## Abstract

TW-37 is a small molecule B cell lymphoma-2 (Bcl-2) homology 3 mimetic with potential anticancer activities. However, the in vivo anti-cancer effect of TW-37 in human oral cancer has not been properly studied yet. Here, we attempted to confirm antitumor activity of TW37 in human oral cancer. TW-37 significantly inhibited cell proliferation and increased the number of dead cells in MC-3 and HSC-3 human oral cancer cell lines. TW-37 enhanced apoptosis of both cell lines evidenced by annexin V/propidium iodide double staining, sub-G_1_ population analysis and the detection of cleaved poly (ADP-ribose) polymerase and caspase-3. In addition, TW-37 markedly downregulated the expression of Bcl-2 protein, while not affecting Bcl-xL or myeloid cell leukemia-1. In vivo, TW-37 inhibited tumor growth in a nude mice xenograft model without any significant liver and kidney toxicities. Collectively, these data reveal that TW-37 may be a promising small molecule to inhibit human oral cancer.

## Introduction

With a development of patient screening tools and a research on pathways involved in tumorigenesis, anti-cancer drug research was developed by converting from chemotherapy inducing cytotoxic responses to target therapy that interferes specific molecules associated with tumorigenesis [1]. Induction of apoptosis by molecular target-based drugs plays a critical role in cancer treatment against various types of cancers [2–4]. The anti-apoptotic Bcl-2 molecules including B-cell lymphoma 2 (Bcl-2) are key regulators of apoptosis by suppressing the activation of proapoptotic Bcl-2 family [5] and these proteins were often over-expressed in many cancers [6, 7]. Recently, several studies showed that small molecules exhibited anticancer activity by targeting Bcl-2 family which is associated with intrinsic pathways of apoptosis [8, 9]. Thus, the development of anti-apoptotic Bcl-2 inhibitors has important implications for cancer treatment and prevention.

TW-37 is a second-generation benzenesulphonyl derivative of gossypol isolated from cotton seeds and roots. It was recently demonstrated that TW-37 attenuated the activation of Bcl-2, Bcl-xL and myeloid cell leukemia-1 (Mcl-1) via directly binding to Bcl-2 homology 3 (BH3) binding groove with high affinity, resulting in the induction of apoptosis [10, 11]. In the case reports of oral cancer patients, anti-apoptotic Bcl-2 family proteins were markedly increased in precancerous lesions suggesting that they can act as oncoproteins for oral carcinogenesis [12–14]. Previously, our group published that BH3 mimetics such as ABT-263 and ABT-737 exhibited apoptosis-inducing potential in oral cancer [15–17]. Recently, we also showed the in vitro anti-cancer effects of TW-37 in human oral cancer cell lines [18]. These suggest that small molecule inhibitors targeting Bcl-2 family proteins can be a promising drug candidate against human oral cancer. To date, however, no study on the in vivo effect of TW-37 in oral cancer has been conducted.

In the present study, we sought to identify in vitro and in vivo anti-cancer activity of TW-37 in human oral cancer.

## Methods

### Cell culture and treatment

The MC-3 mucoepidermoid carcinoma cell line was kindly provided by prof. Wu Junzheng (Forth Military Medical University, Xi’an, China) and HSC-3 oral squamous cell carcinoma cell line was given by Prof. Masanobu Shindoh from Hokkaido University (Hokkaido, Japan). Cells were cultured in Dulbecco’s Modified Eagle Medium/F12 supplemented with 10% heat inactivated fetal bovine serum and antibiotics at 37 °C in a humidified atmosphere 5% CO_2_. All experiments were performed when the cells reached approximately 50~60% confluence. TW-37 (ApexBio, Houston, TX, USA) was dissolved in dimethyl sulfoxide (DMSO), aliquoted, and stored at − 20 °C. Final concentration of DMSO did not exceed 0.1%.

### Trypan blue exclusion assay

The anti-proliferative effect of TW-37 was determined with trypan blue solution (Gibco, Paisley, UK). The number of viable cells, unstained with trypan blue (0.4%) solution, was counted using a hemocytometer with a light microscope.

### Live/dead assay

The cytotoxicity of TW-37 was determined using a Live/Dead Viability/Cytotoxicity assay kit (Life Technologies, Grand Island, NY). The principle of this kit is that live cells are distinguished by the presence of ubiquitous intracellular esterase activity. Briefly, cells were stained with 2 μM calcein-AM and 4 μM ethidium homodimer-1 and incubated for 30 min at room temperature (RT) according to the manufacturer instructions. Cells were then observed using a fluorescence microscopy (Leica DMi8, Wetzlar, Germany).

### Annexin V/PI double staining

Apoptosis was evaluated by double staining with annexin V-fluorescein isothiocyanate (FITC) and propidium iodide (PI), according manufacturer’s instructions of annexin V apoptosis assay kit (BD Biosciences, Franklin Lakes, NJ, USA). The stained cells were analyzed in fluorescence-activated cell sorting (FACS) caliber (Becton-Dickinson) and calculated with Flowjo software (Flowjo LLC, Ashland, OR, USA).

### Cell cycle analysis

Cells were fixed with 70% ethanol overnight at 4 °C. 20 μg/mL (final concentration) of RNase A and PI (P4170, Sigma-Aldrich) were added and then incubated for 15 min at 37 °C. DNA contents were detected using FACS caliber and the relative DNA content was and calculated with FlowJo software.

### Western blotting

After treatment, the cells were harvested, rinsed with phosphate buffered saline (PBS), and then lysed with a lysis buffer on ice. The total protein concentration was measured using a DC Protein Assay Kit (BIO-RAD Laboratories, Madison, WI, USA). The same amount of protein was then loaded onto sodium dodecyl sulfate polyacrylamide gel electrophoresis and transferred onto an immunoblot polyvinylidene fluoride membranes. The membrane was blocked for 2 h in 5% skim milk in tris-buffered saline with Tween 20 and incubated with primary antibodies overnight at 4 °C and corresponding horseradish peroxidase-conjugated secondary antibodies. Antibodies against cleaved poly (ADP-ribose) polymerase (PARP), cleaved caspase-3, Bcl-xL and Mcl-1 were purchased from Cell Signaling Technology, Inc. (Charlottesville, VA, USA) and antibodies against Bcl-2 and β-actin were obtained from Santa Cruz Biotechnology, Inc. (Santa Cruz, CA, USA). Chemiluminescence were detected by SuperSignal West Pico Chemiluminescent Substrate (Santa Cruz Biotechnology Inc.) and were captured by X-ray film or ImageQuant LAS 500 (GE Healthcare Life Sciences, Piscataway, NJ, USA).

### Nude mouse model of human tumor

Six-week-old Balb/c nu/nu male mice (NARA Biotech, Pyeongtaek, Korea) were caged in a facility with a 12-h light/dark cycle and allowed Teklad diet (2018s) and water ad libitum. All mice were handled according to Institutional Animal Care and Use Committee (IACUC) guidelines approved by CHA University (IACUC approval number: 190077). HSC-3 cells were subcutaneously injected into the flanks of the mice and the mice were then assigned randomly to two treatment groups (four mice per group). About 10 days after incubation, tumor-bearing mice were injected intraperitoneally with either vehicle control (PBS) or TW37 (15 mg/kg/day) for 21 days (5 days per week). Tumor volume and was monitored twice a week. Tumor volumes were measured along the two diameter axes with calipers and the tumor volumes were calculated using the equation volume = π/6 × ([D + d]/2)^3^, where D and d are the larger and smaller diameters, respectively. At the termination of the experiment, mice were euthanized, tumors were retrieved to measure tumor weight.

### Histopathological findings

Mouse organs (liver and kidneys) were fixed in 10% neutral buffered formalin. Tissue sections were cut to a thickness of 4 μm and stained with hematoxylin (Mayer’s, Klinipath, Benelux) and eosin (Eosin Y, Klinipath, Benelux). Histopathological changes were analyzed under a Nikon Eclipse E800 microscope.

### Statistical analysis

For the in vitro study, ANOVA followed by Tukey’s post hoc test were used to determine the significance between the control and treatment groups. For the in vivo study, statistical evaluation was performed using the Mann-Whitney U test in SPSS because our data were non-parametric. *p* value of < 0.05 was considered significant (*).

## Results

### Tw-37 inhibits cell growth and viability in MC-3 and HSC-3 human oral cancer cell lines

**Fig. 1:**
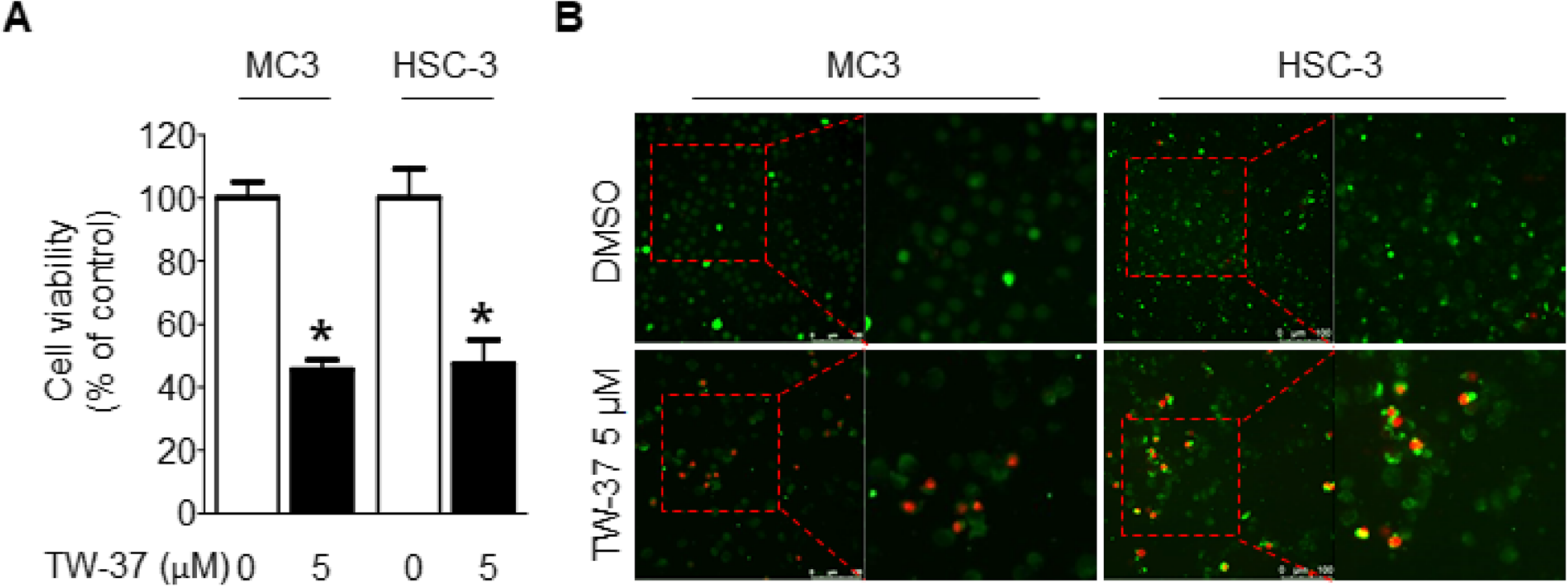
Effect of TW-37 on the viability in human oral cancer cell lines. MC-3 and HSC-3 cell lines were treated with DMSO or 5 μM of TW-37 for 48 h. **a** Cell viability (%) was assessed by trypan blue exclusion assay. The data shown in the graph represent the mean ± SD of triplicate experiments. *, *p* < 0.05 compared with the control. **b** Cytotoxic effect of TW-37 was determined by Live/dead assay kit. Live cells (green fluorescence) and dead cells (red fluorescence) were observed under a light microscope (magnification, × 200)

To explore the anti-proliferative effects of TW-37, MC-3 and HSC-3 cell lines were treated with TW-37 (5 μM) for 48 h and cell growth was evaluated by a trypan blue exclusion assay. Treatment with TW-37 significantly reduced cell proliferation by more than 50% compared with the control (Fig. 1a). Next, we performed a two-color fluorescence assay, which detects esterase activity or cell permeability to confirm the cytotoxic effect of TW-37 in both cell lines. As shown in Fig. 1b, the number of red fluorescence-positive cells by EthD-1 was markedly increased. These results suggest that TW-37 may have anti-proliferative effect by inhibiting cell growth and inducing cell death in human oral cancer cell lines.

### TW-37 increased the apoptotic cell population in human oral cancer cell lines

**Fig. 2:**
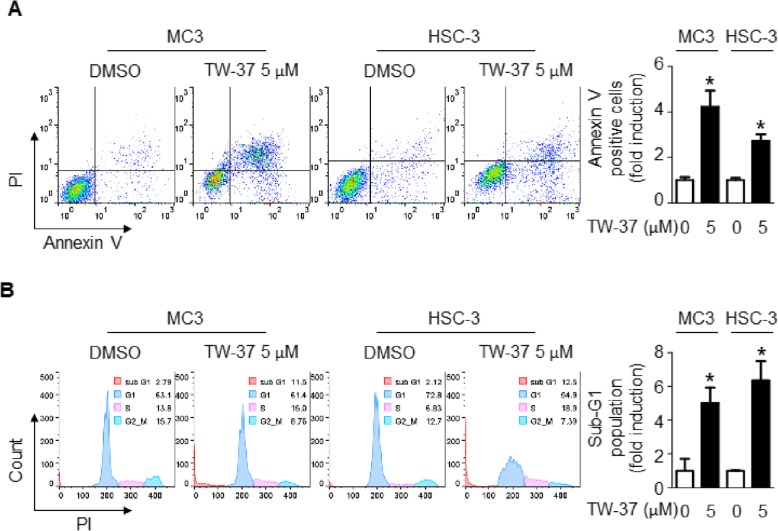
Effect of TW-37 on apoptosis in human oral cancer cell lines. MC-3 and HSC-3 cell lines were treated with DMSO or 5 μM of TW-37 for 48 h. **a** The number of apoptotic cells were determined by annexin V/PI double-staining. **b** Sub-G_1_ population was assessed by FACS analysis. The data shown in the graphs represent the mean ± SD of triplicate experiments. *, *p* < 0.05 compared with the control

To date, many researches revealed that TW-37 can induce apoptotic response in various types of cancer cell lines [16–19]. Thus, we performed annexin V/PI staining and DAPI staining to evaluate the effect of TW-37 in MC-3 and HSC-3 cell lines. The results showed that the percentage of apoptotic cell population (annexin V-FITC-positive) in TW-37- treated group (25.38% for MC-3 cells and 37.00% for HSC-3 cells) dramatically increased compared with in control-treated group (8.74% for MC-3 cells and 13.54% for HSC-3 cells) (Fig. 2a). We further performed cell cycle analysis to determine the sub-G_1_ population. As shown in Fig. 2b, the appearance of sub-G_1_ peak (apoptotic feature) was significantly induced by TW-37 (7.53% for MC-3 cells and 13.70% for HSC-3 cells) compared with control (0.64% for MC-3 cells and 2.15% for HSC-3 cells). These results suggest that TW-37 can induce morphological changes of apoptotic body and fraction of DNA associated with apoptosis in human oral cancer cells.

### TW-37 induced the apoptosis by downregulating Bcl-2 protein in human oral cancer cells

**Fig. 3:**
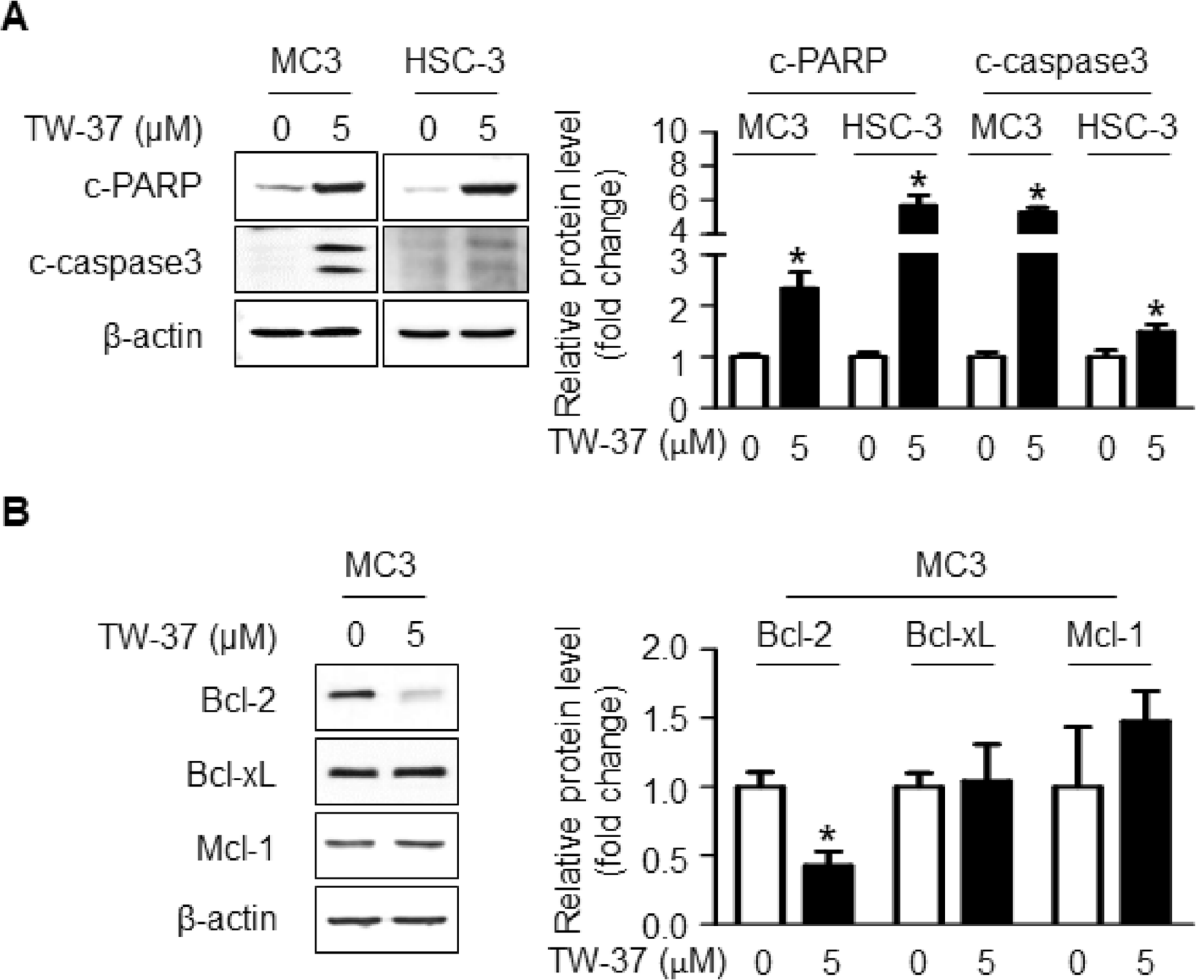
Effect of TW-37 on Bcl-2 family-related apoptosis in human oral cancer cells. **a** and **b** the apoptosis related proteins (c-PARP and c-caspase3) and anti-apoptotic Bcl-2 family proteins (Bcl-2, Bcl-xL, and Mcl-1) were detected using western blot analysis. β-actin was used as a loading control. The data shown in the graphs represent the mean ± SD of triplicate experiments. *, *p* < 0.05 compared with the control

To ascertain TW-37-mediated apoptosis in human oral cancer cell lines, we did western blotting using antibodies against cleaved caspase3 and cleaved PARP, which are known as apoptosis-related protein markers. As shown in Fig. 3a, TW-37 treatment effectively induced apoptotic cell death, as evidenced by the cleavages of caspase 3 and PARP. Because TW-37 is one of BH-3 mimetics that are able to target anti-apoptotic Bcl-2 family proteins, we investigated whether TW-37 affects those proteins such as Bcl-2, Bcl-xL, and Mcl-1. As shown in Fig. 3b, Bcl-2 expression was significantly reduced by TW-37 while not affecting Bcl-xL and Mcl-1 protein. These results suggest that TW-37-induced apoptosis may be related with the downregulation of Bcl-2 protein.

### TW-37 suppresses tumor growth in a tumor xenograft model without any toxicity

**Fig. 4:**
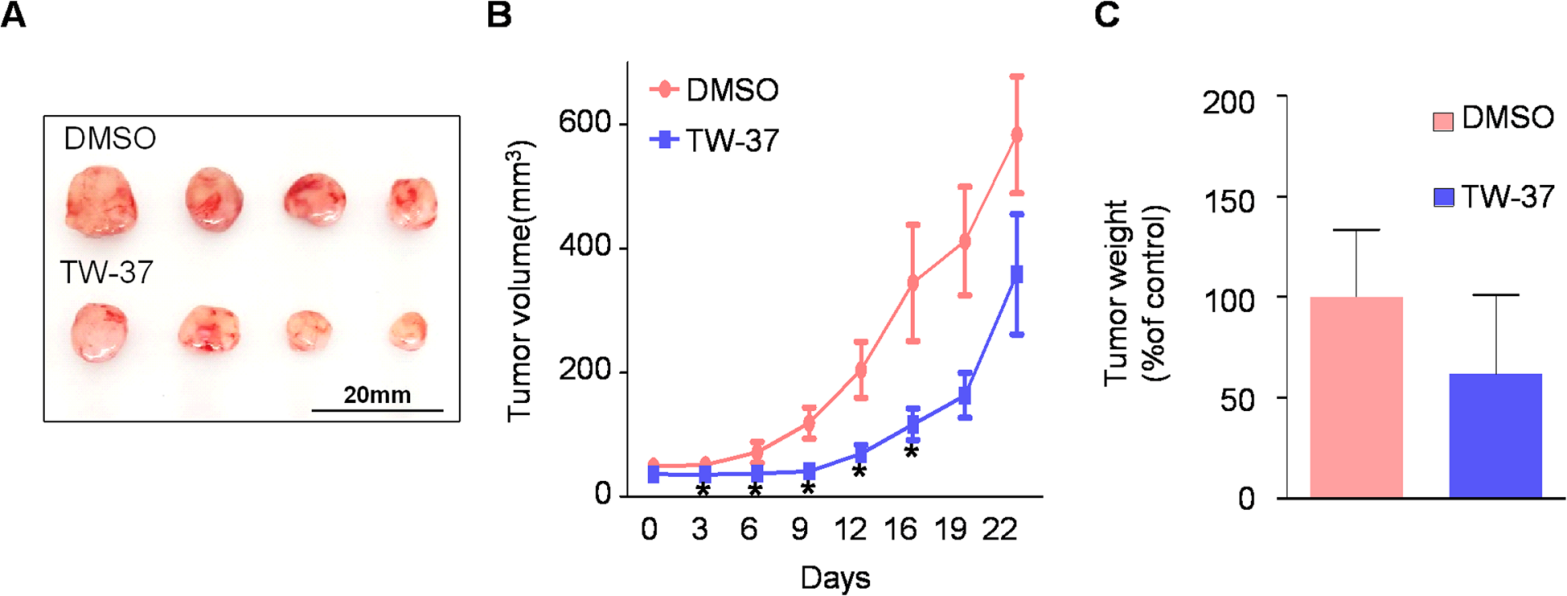
Effect of TW-37 on tumor growth in a xenograft mice model. Balb/c nude baring HSC-3 cells were treated with vehicle control (PBS) or TW-37 (15 mg/kg/day) for 21 days (*n* = 4/group). **a** The photograph of tumor tissues. Tumor volume (**b**) and tumor weight (**c**) were measured. The values are presented as mean ± SEM of 4 mice per group. *, *p* < 0.05 compared with the control

**Fig. 5:**
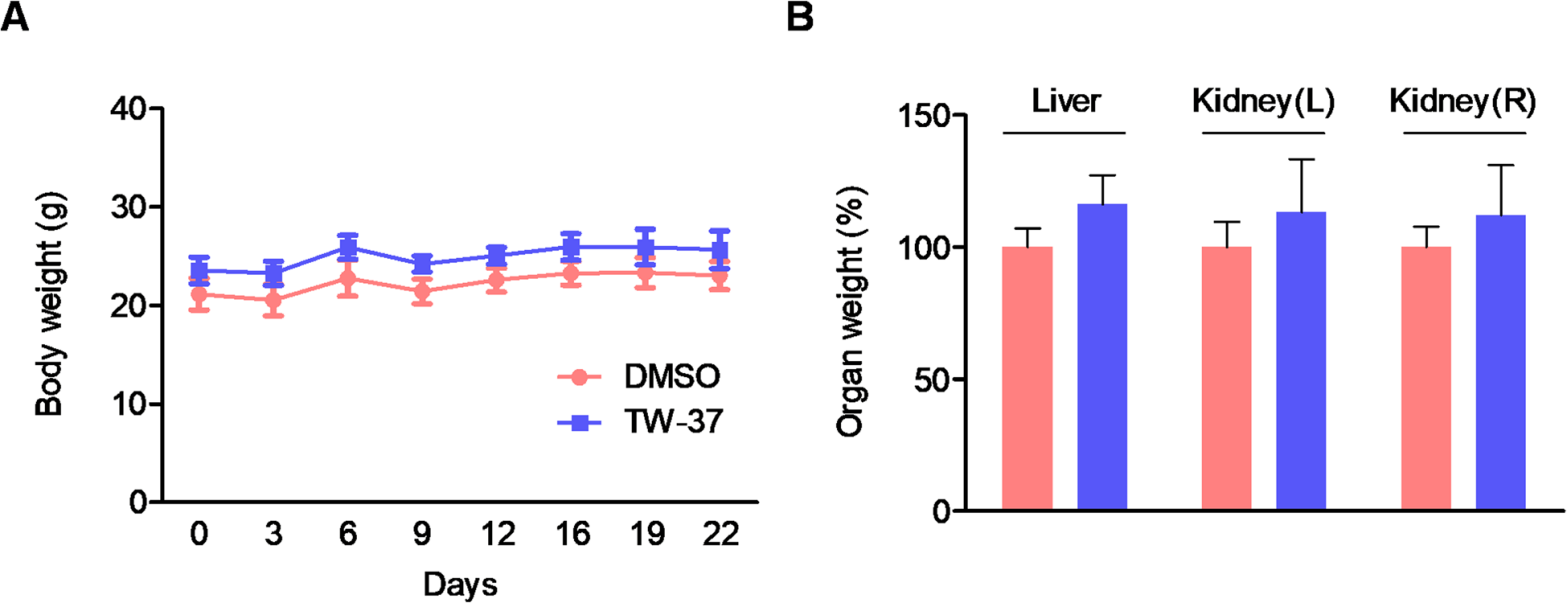
Effect of TW-37 on body and organs weight. Body weight (**a**) and weights of liver and kidney (**b**) were measured after sacrifice. Each graph represents mean ± SEM of 4 mice per group

**Fig. 6:**
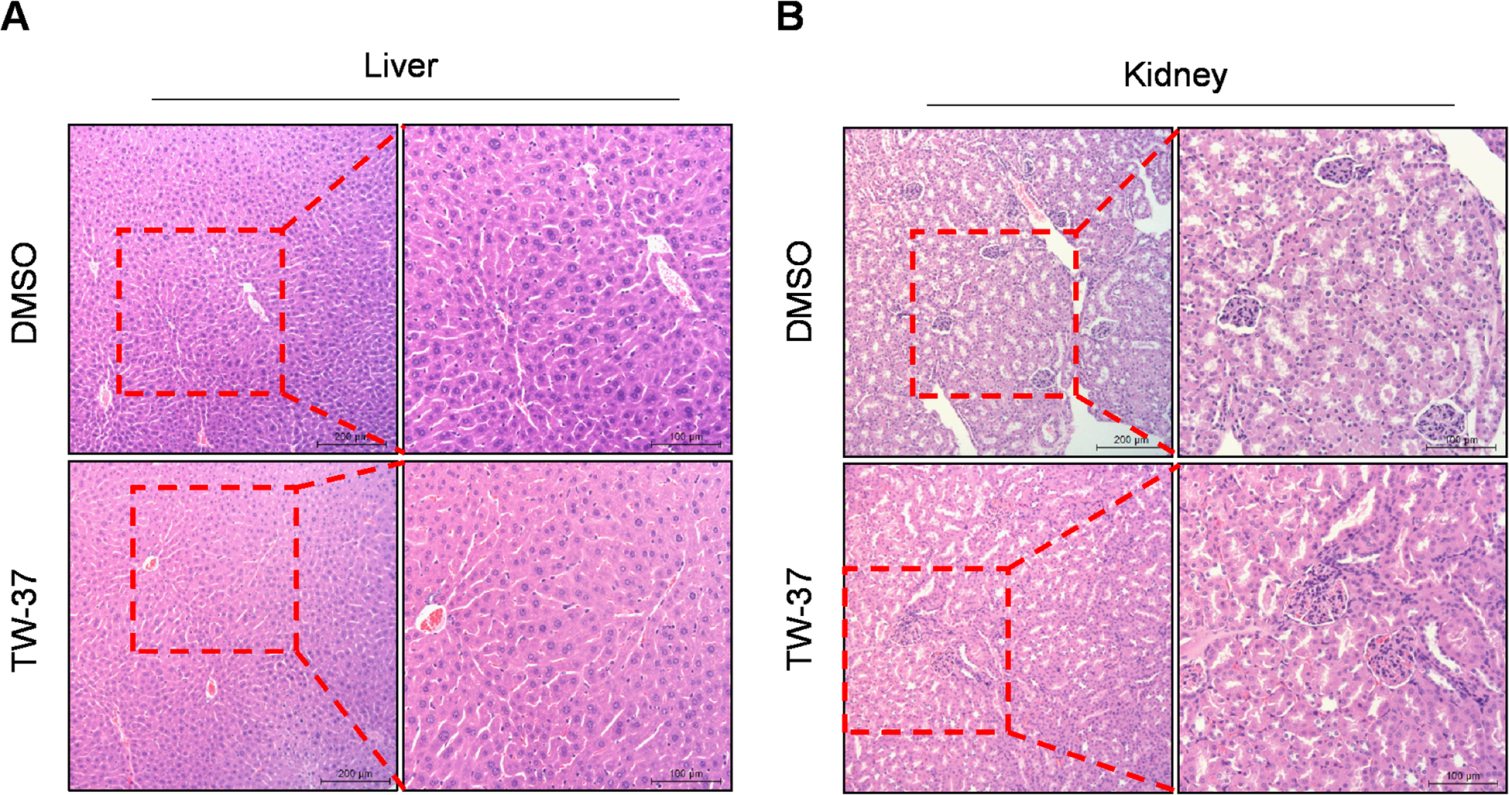
Histopathological findings of liver (**a**) and kidney (**b**). (magnification, × 100)

To evaluate the efficacy of in vivo antitumor activity of TW-37, we used a xenograft mice model by subcutaneously injection of HSC-3 cells into the flank of athymic nude mice. As shown in Fig. 4a and b, TW-37 treatment significantly decreased tumor volume on day 16 after the treatment. Consistent with this, the average of tumor weight was reduced at a very slight trend toward significance (Fig. 4c). Next, to investigate the effects of TW-37 on in vivo biocompatibility, we measured the body and organs (liver and kidneys) weights. TW-37 treatment did not affect significant changes of body and organs weight (Fig. 5). In addition, histopathologic evaluation of liver and kidney did not show any pathological differences between the control- and TW-37-treated mice (Fig. 6). These results suggest that TW-37 administration suppresses tumor growth in our mouse xenograft model of human oral tumor without causing histopathological toxicity.

## Discussion

Apoptosis is the most important physiological cell death and is tightly regulated by pro- and antiapoptotic cascades during development and aging. Homeostatic state between apoptosis and cell division is essential for maintaining cell populations in normal tissues. Thus, the dysregulation of apoptosis can cause many human diseases including cancer [19, 20]. A number of small molecule inhibitors were previously designed to induce apoptosis against cancers by targeting anti-apoptotic Bcl-2 proteins characterized into groups based on their structure and function [21]. For instance, venetoclax (ABT-199/GDC-0199) is typical Bcl-2 inhibitor that plays a role as BH3 mimic for the treatment of Bcl-2-dependent hematological cancers [22]. Lin et al. [23] also reported that ABT-263 induced cell cycle arrest, apoptosis and autophagy in human esophageal cancer cells.

In 2006, TW-37 was developed as a small molecule inhibitor of Bcl-2, Bcl-XL, and Mcl-1 and was shown to have anti-cancer activity in lymphoma cells [24]. Several researches showed that TW-37 stimulated apoptotic cell death and changed cytotoxicity towards the drug-sensitive and resistance in nasopharyngeal carcinoma, colorectal cancer, ovarian cancer, and neuroblastoma [25–28]. In this study, we investigated the anti-cancer activity of TW-37 in MC-3 and HSC-3 human oral cancer cell lines. Consistent with the discovery of others, TW-37 significantly exhibited anti-cancer activity in human oral cancer cells via the induction of apoptosis. In addition, we found that TW-37 specifically affected Bcl-2 proteins suggesting that Bcl-2 is a major molecule target for TW-37-induced apoptosis as expected. In contrast, our group recently published that lower concentration (1.25 μM) of TW-37 did not inhibit cell growth and induce apoptosis in MC-3 cells [18]. It means that the findings of the current study do not support our previous research. A possible explanation for this might be that only higher concentration of TW-37 may be associated with Bcl-2 protein during its apoptotic activity in MC-3 cells. However, future investigations might explain why only higher concentration of TW-37 may act as a Bcl-2 inhibitor.

Several reports have shown that TW-37 treatment significantly inhibited tumor growth in vivo without any toxicity or any loss of body weight [25, 29]. However, there have been no studies using experimental animals for human oral cancer. In the present study, we found that TW-37 also suppressed tumor growth without any body weight loss and any histopathologic changes of both liver and kidney tissues (Figs. 4, 5 and 6). These data broadly support the work of other studies. However, one unanticipated finding was that it was only significantly effective until the day 16 of TW-37 material treatment and its antitumor activity was not significant at the time of sacrifice (Fig. 4b). It seems possible that these results may be due to the acquisition of drug resistance to TW-37 in MC-3 cells. However, we still need to determine how MC3 cells acquire drug tolerance in the early time. Innate or acquired resistance to chemotherapy is a major cause of treatment failure in cancer patients. Despite BH3 mimetics targeting members of Bcl-2 family have received much attention for the recent success of cancer treatments, particularly hematological malignancies, single drug BH3 mimetic therapy also has limited effectiveness [30, 31]. Actually, there are such previous studies showing that BH3 mimetics had drug resistances by compensatory activity of Mcl-1 for the inhibition of Bcl-2/Bcl-xL and Mcl-1 activation sensitized cancer cells to BH3 mimetics [32–35]. These suggest that the combinational therapeutic strategy could directly activate the cell death mechanism to overcome its limitation. Thus, it is worthwhile to clarify it in future studies although combination strategy with TW-37 remains unclear.

## Conclusions

The study presented here showed the anti-cancer effects of TW37 in human oral cancer cell line and nude mice tumor xenograft model. Our findings provide a strong possibility of TW-37 as a potential antitumor agent for oral cancer despite several important questions still need to be explained.

## Data Availability

The datasets used and/or analyzed in this study are available from the corresponding author on reasonable request.

## Abbreviations

Bcl-2: B cell lymphoma-2
BH3: Bcl-2 homology 3
Mcl-1: Myeloid cell leukemia-1
PARP: Poly (ADP-ribose) polymerase
